# The molecular profile in patients with polycythemia vera and essential thrombocythemia is dynamic and correlates with disease’s phenotype

**DOI:** 10.3389/fonc.2023.1224590

**Published:** 2023-08-21

**Authors:** Patryk Sobieralski, Bartosz Wasąg, Aleksandra Leszczyńska, Monika Żuk, Maria Bieniaszewska

**Affiliations:** ^1^ Department of Hematology and Transplantology, Medical University of Gdansk, Gdansk, Poland; ^2^ Department of Biology and Medical Genetics, Faculty of Medicine, Medical University of Gdańsk, Gdansk, Poland; ^3^ Laboratory of Clinical Genetics, University Clinical Centre, Gdansk, Poland

**Keywords:** polycythemia vera, essential thrombocythemia, molecular profile, thrombosis, secondary myelofibrosis, next-generation sequencing

## Abstract

**Introduction:**

Polycythemia vera (PV) and essential thrombocythemia (ET) are diseases driven by canonical mutations in JAK2, CALR, or MPL gene. Previous studies revealed that in addition to driver mutations, patients with PV and ET can harbor other mutations in various genes, with no established impact on disease phenotype. We hypothesized that the molecular profile of patients with PV and ET is dynamic throughout the disease.

**Methods:**

In this study, we performed a 37-gene targeted next-generation sequencing panel on the DNA samples collected from 49 study participants in two-time points, separated by 78-141 months. We identified 78 variants across 37 analyzed genes in the study population.

**Results:**

By analyzing the change in variant allele frequencies and revealing the acquisition of new mutations during the disease, we confirmed the dynamic nature of the molecular profile of patients with PV and ET. We found connections between specific variants with the development of secondary myelofibrosis, thrombotic events, and response to treatment. We confronted our results with existing conventional and mutation-enhanced prognostic systems, showing the limited utility of available prognostic tools.

**Discussion:**

The results of this study underline the significance of repeated molecular testing in patients with PV and ET and indicate the need for further research within this field to better understand the disease and improve available prognostic tools.

## 1 Introduction

Myeloproliferative neoplasms (MPN) are driven by specific mutations in one of the three genes – *JAK2, MPL* or *CALR* (1). These mutations cause uncontrolled activation of the JAK-STAT signaling pathway leading to the release of secondary mediators of pro-survival and proliferative nature (2).

The rapid development of sequencing techniques in recent years allowed for better characterization of molecular profiles in patients with hematological malignancies and significantly contributed to an understanding of disease biology. It is accepted that besides driver mutations, the molecular profile of MPN comprises variants in a variety of genes (3, 4). The presence and allelic burden of those mutations are suspected to impact the disease’s clinical course. However, in MPNs, the chronic nature of the disease hinders the research aimed at understanding the significance of molecular changes, contrary to entities of acute nature. Mutations in only a fraction of genes have been recently proposed as potentially influencing disease risk and included in mutation-enhanced international prognostic systems (MIPSS) for polycythemia vera (PV) and essential thrombocythemia (ET) (5).

In this study, we aim to assess the dynamics of the molecular profile in PV and ET by investigating the occurrence and allelic burden of both driver and passenger mutations at two-time points. We hypothesize that the molecular profile of patients with PV and ET evolves with time, accumulating mutations in expanding malignant clones. The chronic nature of those diseases implies the need to assess the risk multiple times. We believe studies on the topic may help predict patient outcomes in advance, giving the time to react and treat accordingly.

## 2 Patients and methods

Study participants have been selected from the population of patients of the Outpatient Ward, Department of Hematology and Transplantology, Medical University of Gdansk, Poland. Initially, all alive patients diagnosed with PV or ET were identified. Next, individuals for whom the diagnosis was made at least five years prior to the study were selected. Among those patients, we identified individuals from whom DNA samples of sufficient quality and quantity were available from the time of diagnosis. Finally, 49 patients consented to participate in the study.

Diagnoses were verified with MPN 2016 WHO criteria (6). Two patients with apparent features of myeloproliferative neoplasms but undetected driver mutation were labeled triple-negative (TN). Each patient’s documentation was analyzed to collect data regarding treatment, thrombotic complications, and disease progression. The general characteristics of the study group are presented in **Table 1**.

**Table 1:** General characteristics of the study group.

	1st sample	2nd sample
PV		
**N**	13	
**Sex, F/M**	7/6	
**Sample collection, months (range)**	Diagnosis	104 months (83 -134)
**Age, years, median (range)**	57 (24 - 68)	66 (34 - 77)
**Hb, g/dl, median (range)**	17,9 (14,5 - 22)	14,9 (12 – 16,7)
**PLT, G/l, median (range)**	507 (315 - 906)	319 (181 – 744)
**WBC, G/l, median (range)**	9,49 (6,26 - 21,12)	5,92 (4,04 – 26,71)
**LDH, U/L, median (range)**	186 (147 – 442)	221 (146 – 796)
**Cytoreduction/Observation**	11/2	13/0
**Treatment change (%)**	n/a	3 (23)
**Thrombosis (%)**	3 (23)	2 (15)
**Risk low/high**	6/7	n/a
**MIPSS low/int/high**	11/2/0	5/8/0
**Progression to MF (%)**	n/a	2 (15)
**Progression to AML (%)**	n/a	0
ET		
**N**	36	
**Sex, F/M**	23/13	
**Sample collection, months (range)**	Diagnosis	104 months (78-141)
**Age, years, median (range)**	54 (28 – 67)	63 (35 – 74)
**Hb, g/dl, median (range)**	13,9 (10 – 16,4)	13,5 (8,8 – 17)
**PLT, G/l, median (range)**	760 (516 – 1750)	425 (22 – 824)
**WBC, G/l, median (range)**	7,9 (4,9 – 25,9)	6,5 (3,4 – 19,3)
**LDH, U/L, median (range)**	209 (129 – 473)	222 (128 – 1056)
**Cytoreduction/Observation**	28/8	32/4
**Treatment change (%)**	n/a	17 (47)
**Thrombosis (%)**	2 (5)	4 (11)
**Risk low/high**	26/10	n/a
**MIPSS low/int/high**	27/5/4	13/12/11
**Progression to MF (%)**	n/a	7 (19)
**Progression to AML (%)**	n/a	0

n/a, not applicable.

A peripheral blood sample was collected from each participant for molecular testing and comparison with corresponding archival samples. The median time between historical sample collection and a present sample was 104 months (range 78-141 months)

Genomic DNA was extracted from peripheral blood samples using QIAamp DNA mini kit (Qiagen) according to the manufacturer’s instructions. DNA was quantified by spectrophotometric method and stored at –20°C for further analysis.

Next-generation sequencing was performed using the Archer VariantPlex Core Myeloid kit (ArcherDX), Mid Output Kit (300-cycles), and MiniSeq (Illumina). It allowed performing a comprehensive analysis of 37 genes: *ABL1, ANKRD26, ASXL1, BCOR, BRAF, CALR, CBL, CABPA, CSF3R, DDX41, DNMT3A, ETNK1, ETV6, EZH2, FLT3, GATA1, GATA2, IDH1, IDH2, JAK2, KIT, KRAS, MPL, NPM1, NRAS, PHF6, PTPN11, RUNX1, SETBP1, SF3B1, SRSF2, STAG2, TET2, TP53, U2AF1, WT1, ZRSR2*. The results were analyzed using Archer Analysis v.6.0.3.2 software (ArcherDX), and 2,7% allele frequency (VAF) as a cutoff was applied.

Collected data was used to stratify patients into the risk groups during sample collection. Conventional risk stratification was made according to the revised IPSET-T system in ET and based on age and thrombotic complications in PV (7, 8). Acquired sequencing data was used to attribute patients into the low- intermediate- or high-risk groups according to MIPSS-ET or MIPSS-PV (5). Since cytogenetic data were incomplete in the study population, all PV patients were considered to have normal karyotypes.

An additional exploratory risk stratification, including 5- and 10-year event-free survival (EFS), risk of secondary myelofibrosis (sMF) or acute myeloid leukemia (AML) were assigned based on prognostication model developed by Grinfeld et al. (4). This stratification was made to assess the utility of the proposed MPN Personalized Risk calculator (available online at https://www.sanger.ac.uk/science/tools/progmod/progmod/). For this evaluation, all patients were considered unknown karyotypes.

Statistical analysis was performed using the computer program STATISTICA v.13.3. Data was statistically described in terms of mean or median and range. Comparison between the two groups was made using Mann–Whitney U test for continuous variables and the Chi-square test for categorical variables. P values were considered significant if less than 0.05.

## 3 Results

### 3.1 General findings

**Figure 1:**
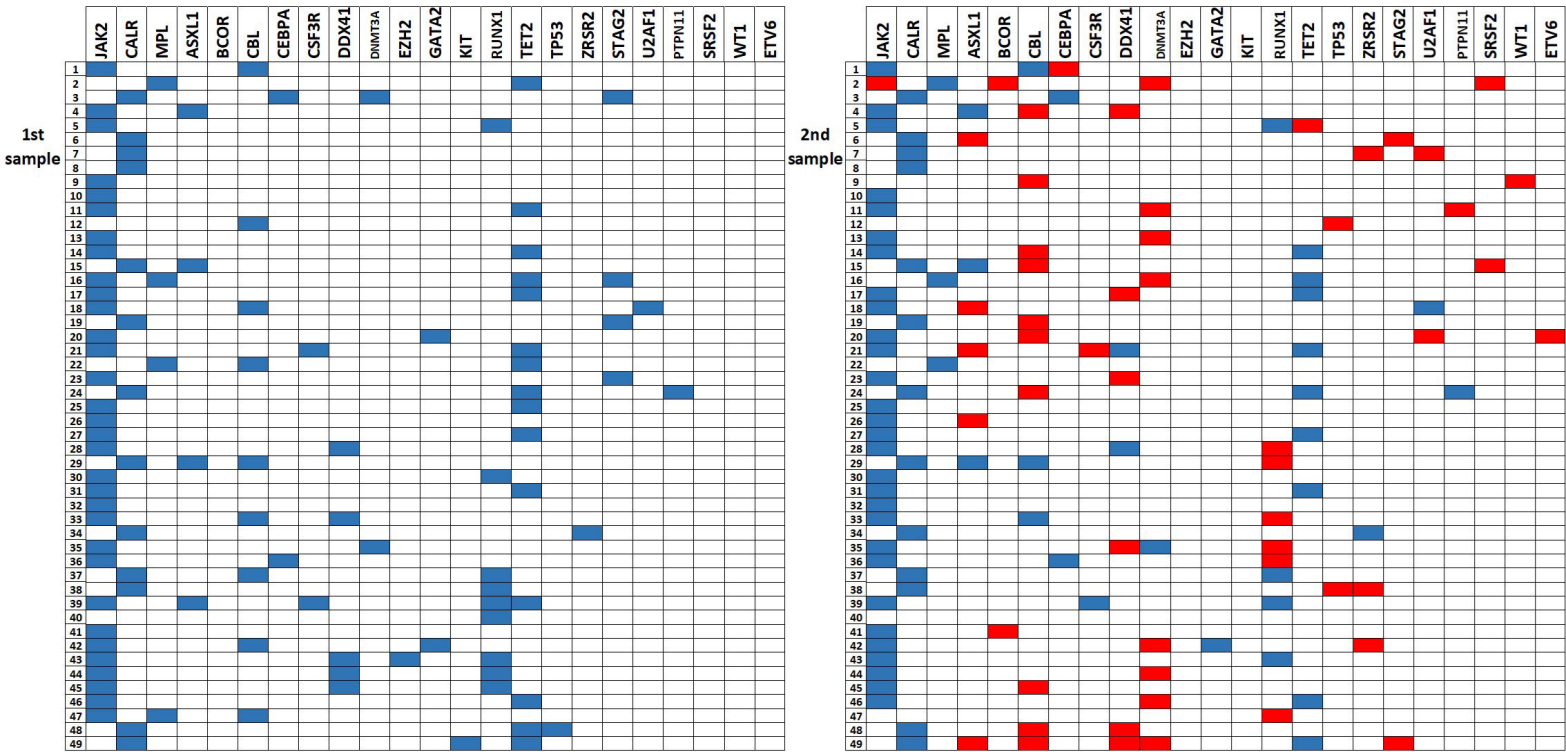
The gene plot showing detected mutations in analyzed genes across 1^st^ and 2^nd^ samples in each patient. New mutations in 2^nd^ sample are highlighted in red.

**Figure 2:**
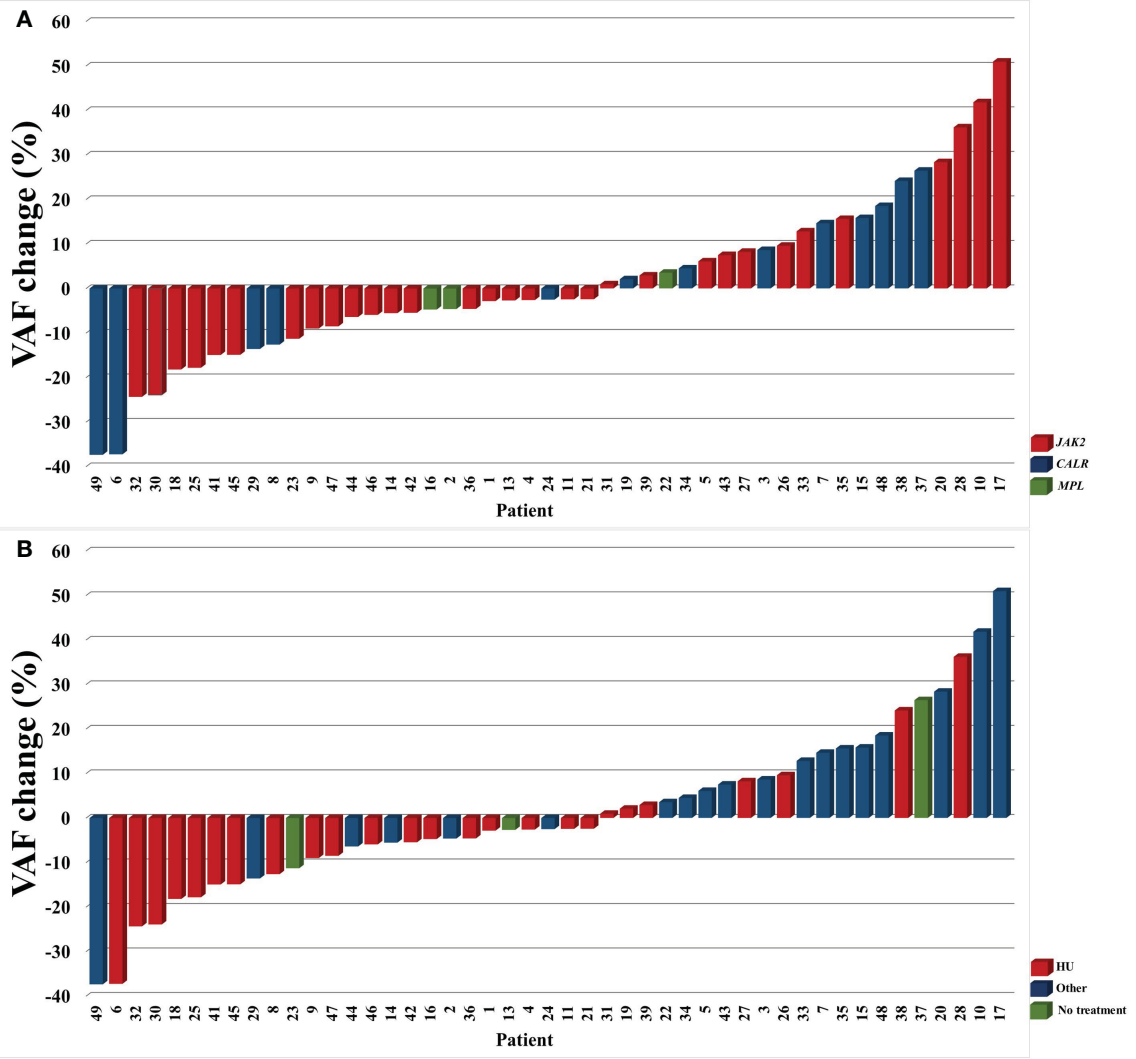
The dynamics of VAF of driver mutations in analyzed subgroups by the type of driver mutation **(A)** and treatment **(B)**. TN patients (#12 and 40) are not included.

A total of 98 samples were analyzed, including 49 archival samples and 49 follow-up samples. In summary, 78 variants were detected among the analyzed 37 genes (**Supplementary Table 1**). The most frequently detected variants in driver genes were *JAK2* p.Val617Phe (32 pts), *CALR type 1* (8 pts), *CALR type 2* (5 pts) and *MPL* p.Trp515Leu (2 pts). The gene plot showing detected mutations in analyzed genes across 1^st^ and 2^nd^ samples of each patient is presented in **Figure 1**. New variants detected on the 2^nd^ sample are highlighted in red. The dynamics of VAF of driver mutations is shown in **Figure 2A**.

In contrast with the paradigm that driver mutations in MPNs are mutually exclusive, our analysis revealed two coexisting canonical mutations in one patient (#16) – *MPL* p.Trp515Leu variant (VAF 14,02%) and *JAK2* p.Val617Phe variant (VAF 5,17%) at diagnosis. In the follow-up sample, only the *MPL* p.Trp515Leu variant was detectable (VAF 9,3%).

Non-canonical variants in driver genes were detected in four patients. One patient (#2) had non-canonical driver mutation in *MPL* gene – p.Ser505Asn c.1514G>A – present at diagnosis and follow-up, with VAF of 31,92% and 27,31%, respectively, and coexisting with *JAK2* p.Val617Phe, detectable only in a second sample at relatively low VAF (6,47%). In the remaining three patients, non-canonical variants in driver genes also coexisted with canonical mutations and included *JAK2* p.Lys607Asn c.1821G>T detected along with *JAK2* p.Val617Phe (#14), *MPL* c.1565 + 5C>T detected along with *JAK2* p.Val617Phe (#47) and *MPL* p.Ser493Phe c.1478C>T was observed along with *MPL* p.Trp515Leu (#22). No non-canonical mutations in *CALR* were detected.

In three female ET patients receiving hydroxyurea monotherapy (#9, 16, and 47), *JAK2* p.Val617Phe mutation was detectable at diagnosis but not at follow-up. Here, the variant was initially detected at relatively low VAF (5,17-8,9%) and probably reduced below the detection threshold at follow-up (<2,7%). Several other mutations were undetectable in follow-up samples (**Figure 1**; **Supplementary Table 1**). Those variants were detected at low VAFs at diagnosis and did not have confirmed reports of pathogenicity.

Among TN patients (#12 and 40) we detected variants with low VAFs, including CBL p.Asp460del c.1380_1382del variant (VAF 2,75%), RUNX1 p.Arg423Gly c.1267C>G (VAF 3,72%) variant and pathogenic TP53 p.Arg248Leu c.743G>T (VAF 6,96%) variant.

Among non-driver genes, the most frequently detected variants were *CBL* p.Asp460del c.1380_1382del (19 patients), *RUNX1*p.Glu422Ala c.1265A>C (11 patients), *DDX41* p.Val408Asp c.1223T>A (9 patients) and *STAG2* p.Glu342Ter c.1024G>T (5 patients). Those variants have not established pathogenicity.

**Figure 3:**
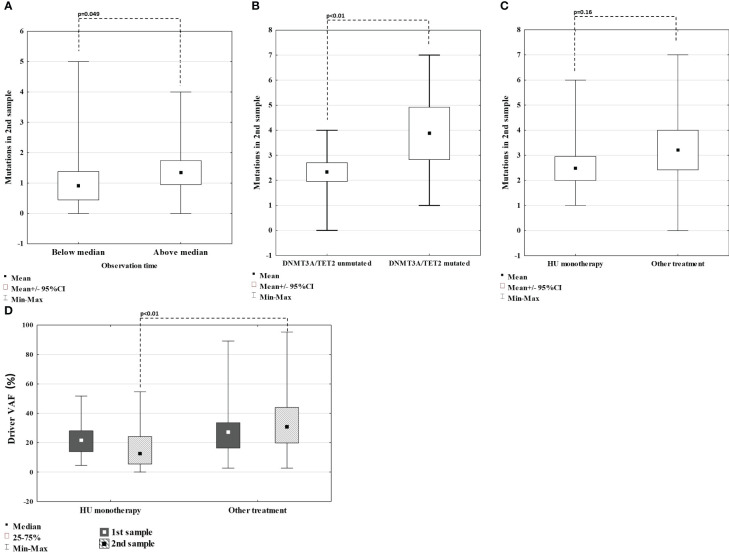
Comparison of number of mutations detected on 2^nd^ sample in regard of time between sample collection **(A)**; in patients with and without *TET2* or *DNMT3A* mutation **(B)**; in patients treated with HU monotherapy **(C)**. Comparison of VAF of driver mutation in 1^st^ and 2^nd^ sample in patients treated with HU monotherapy vs others **(D)**.

Since in our study population, the time to the 2^nd^ sample acquisition ranges from 78 to 141 months (median 104 months), we wanted to investigate whether there is a difference in the number of variants detected on 2^nd^ sample in patients with longer observation time (above median) compared to patients with shorter observation time (below median). The mean number of variants was higher in patients with longer observation times, with a borderline significance of p=0.049 (**Figure 3A**). We also performed the analysis to verify whether some variants detected in the second sample correlate with disease progression. A number of variants detected in both diagnosis and follow-up samples were similar in patients requiring treatment change, experiencing thrombotic complications and developing fibrotic progression (p>0.05 for all comparisons).

The two most frequently mutated genes were *TET2* (18 variants) and *DNMT3A* (ten variants). We observed a high prevalence of *TET2* mutations, both in diagnosis (n=15), with median VAFs of 46,99% (range 3,49 – 54,94%), and follow-up samples (n=10), with median VAFs of 49,02% (20,06 – 50,09%), suggesting its germline origin. Patients harboring *TET2* mutation in 1^st^ sample were older (median of 59 years, compared to 54 for the rest of the group). However, this difference was not statistically significant (p=0.12). On the other hand, *DNMT3A* mutations were rarely detected at diagnosis (n=2) with median VAFs 9,22% (8,03 – 10,41%) but frequently at follow-up samples (n=9) with median VAFs of 4,7% (3,53 – 25,01%), corresponding with somatic origin. Patients that acquired *DNMT3A* mutations had median time between sample collection of 102 months, below the median for the whole group. Moreover, patients with *DNMT3A* mutation emerging in the second sample (n=8), had median age of 64 years, compared to 65 years for the rest of the group. Those findings indicate that acquiring DNMT3A mutations is not connected with the duration of treatment or age. Next, we confronted whether specific patterns of *DNMT3A/TET2* mutation dynamics correlated with the clinical picture. *DNMT3A/TET2* mutations were rarely observed in patients developing sMF (at diagnosis none with *DNMT3A* and four with *TET2*, at follow-up one with *DNMT3A* and two with *TET2*). Four out of eight emerging *DNMT3A* mutations were detected in patients receiving hydroxyurea (HU) monotherapy. On the other hand, VAFs of *TET2* mutations were reduced (-0,18% to -19,84%) in patients on HU monotherapy. There were no differences in the presence or VAF of *DNMT3A/TET2* mutations in patients requiring treatment change or experiencing thrombosis compared to others. However, patients harboring *TET2* or *DNMT3A* mutations in the first sample had significantly more variants overall detected in the second sample, compared with *TET2* or *DNMT3A-*unmutated patients (**Figure 3B**). Additionally, one patient (#35) harbored *DNMT3A* p.Glu733Ala variant with VAF increasing across measurements from 10% to 23%, significantly higher than of other *DNMT3A* variants detected and this variant was previously connected with progression to AML.

During the study period, we observed emerging *ASXL1* mutations in five patients. Three patients (#18, 21, 49) acquired the p.Gly646TrpfsTer12 variant which was previously described in myelofibrosis and AML. Patient #18 developed myelodysplasia, and #49 developed sMF. Other variants included p.Pro808LeufsTer10 c.2421del (#26), previously described in MDS patients with fibrosis, and p.Thr736GlnfsTer8 (#6), undescribed previously.

### 3.2 Risk groups

Using the revised IPSET-T stratification system in ET patients at diagnosis, 26 were stratified to the non-high-risk group (very low - 10 patients, low - 16 patients), whereas 10 patients were attributed to the high-risk group. Among the high-risk patients, two had thrombotic complications (both myocardial infarction), and eight were above or equal to 60 years of age.

Using the conventional risk stratification in PV patients at diagnosis, six were attributed to the low-risk group, whereas seven were attributed to the high-risk group. Among high-risk patients three had thrombotic complications (two ischemic strokes, one deep vein thrombosis) and four were above or equal to 60 years of age.

The second assessment was performed by calculating the risk according to MIPSS-ET and MIPSS-PV at each sample collection. We also checked in which cases the MIPSS score was increased by findings from genetic analysis. In most patients, the MIPSS score increase between two consecutive samples was mediated only by age (17 patients in ET, six patients in PV). In the ET group, results from the genetic analysis on the 2^nd^ sample placed the patient in the high-risk group in as many as five cases. On the contrary, no such observation was made in the PV group, even though one young patient acquired a known pathogenic mutation in the *TP53* gene (p.Arg248Leu c.743G>T).

**Figure 4:**
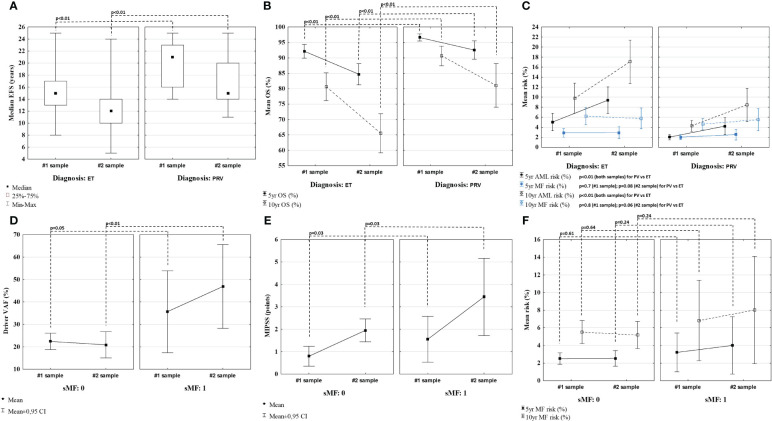
Comparison of event free survival (EFS) **(A)**, overall survival (OS) **(B)**, and 5- and 10-year risk of MF/AML **(C)**, calculated using MPN Personalized Risk Calculator using the data from the time of each sample collection. Comparison of driver mutation VAF **(D)**, score attributed by MIPSS **(E)** and MF risk **(F)** in patients with and without secondary myelofibrosis (sMF).

The final risk stratification was made using MPN Personalized Risk Calculator. Patients with PRV had significantly higher calculated median EFS (p<0.01), 5- and 10-year OS (p<0.01 for both), and lower 5- and 10-year AML risk (p<0.01 for both), both at diagnosis and follow-up when compared to ET patients. On the other hand, 5- and 10-year MF risk calculated at diagnosis and follow-up was comparable between ET and PV patients (p=0.7; p=0.8 and p=0.8; p=0.6, respectively). Results are presented in **Figures 4A–C**.

### 3.3 Treatment

At diagnosis, 38 (78%) patients were started with cytoreduction with HU, one patient (young female) received interferon, while ten patients were without cytoreductive treatment. During the time between each sample collection, 20 patients required treatment change, while 25 patients remained on treatment with HU. The reasons for treatment change were refractoriness to previous therapy (nine patients), the toxicity of prior treatment (seven patients), progression to sMF (two patients) and reaching the conventional high-risk group (two patients). At follow-up, patients were treated with various cytoreductive agents, including hydroxyurea, busulfan, anagrelide, peg-IFN-a2a, and ruxolitinib. Four patients were not receiving cytoreduction at follow-up - one patient with sMF treated symptomatically with blood transfusions and three patients who were not started with cytoreduction from the diagnosis.

Compared to other patients, patients receiving HU monotherapy across the study observation time had similar rates of developing sMF and thrombotic complications, a similar number of new mutations, mutations overall, and *DNMT3A/TET2* mutations detected on 2^nd^ sample (p>0.05 for all comparisons) (**Figure 3C**). Out of five patients who acquired variants in the *ASXL1* gene at follow-up, four (80%) were treated with HU monotherapy (#6, 18, 21, 26). However, patients treated with HU had lower median VAF of driver mutation assessed in the 2^nd^ sample when compared with other patients (p<0.01) (**Figure 2B**; **Figure 3D**). This group also included three patients (#9, 16, and 47) in whom *JAK2* Val617Phe was eliminated in 2^nd^ sample (VAF <2,7%).

We also wanted to look closer at the three patients who endured to be treatment free from the time of diagnosis (#13, 23, 37). None of them experienced thrombotic complications or sMF. All of them were diagnosed with ET. Two of them had *JAK2* p.Val617Phe mutation with VAF on a relatively stable low level between the two measurements (range 8,13-11,8%). The third patient (#37) had *CALR* type 1 mutation with increasing VAF from 25,17% to 51,63% (**Figure 2**). Patients without treatment had a mean of 0.7 mutations detected in the second sample (one in two patients, none in one patient) compared to a mean of 1.7 mutations (range 0-5) in the rest of the group. Among non-driver mutations, those patients harbored *STAG2* p.Glu342Ter c.1024G>T with VAF of 3,08% (likely benign), *CBL* p.Asp460del c.1380_1382del with VAF of 2,92% (no confirmed pathogenicity), *RUNX1* p.Arg423Gly c.1267C>G with VAF of 2,87% (no confirmed pathogenicity), *RUNX1* p.Glu422Ala c.1265A>C with VAF of 23,15% (benign) at 1^st^ sample, and *DNMT3A* p.Gly109Ala c.326_327inv with VAF of 3,53% (undescribed), *DDX41* p.Val408Asp c.1223T>A with VAF of 4,10% (undescribed, detected frequently in this study), and *RUNX1* p.Glu422Ala c.1265A>C with VAF of 31,11% (benign) at 2^nd^.

### 3.4 Fibrotic progression

**S**even ET (#2, 7, 10, 20, 39, 41 and 48) and two PV (#5 and 27) patients experienced fibrotic progression at follow-up. When compared to patients not developing fibrosis, patients with sMF had similar VAF of driver mutation at diagnosis (p=0.05) but significantly higher at follow-up (p<0.01) (**Figures 4A, D**). Patients with sMF had higher total points attributed to MIPSS, than those without fibrotic progression at diagnosis and follow-up (p=0.03 for both) (**Figures 4B, E**). Using MPN Risk Calculator and based on the data from the diagnosis, patients who later developed sMF during the disease had similar calculated 5- and 10-year MF risk (p=0.61 and p=0.64, respectively) as patients without fibrosis. Moreover, when the risk was assessed based on the results from the follow-up sample, when patients were in fact after or during fibrotic progression, the calculated 5- and 10-year risk of developing sMF was also comparable between patients with and without sMF (p=0.24 for both) (**Figures 4C, F**).

Since we identified a relatively high number of patients developing bone marrow fibrosis during our study, we wanted to investigate further specific variants detected in those patients. To assess the pathogenicity, we decided to distribute those findings into three groups – variants detected both at diagnosis and at follow-up, variants appearing in patients with sMF (not detectable at diagnosis) and variants detectable only at diagnosis (not detectable at follow-up) (**Supplementary Table 2**). The major findings in this group include the detection of *ASXL1* p.Gly646TrpfsTer12 (variant described in MF and connected with AML progression), *RUNX1* p.Leu56Ser and ZRSR2 p.Arg169Ter (variants described in MPN and MDS and connected with fibrotic progression) and U2AF1 p.Gln157Pro (described in MDS, MPN/MDS, MF and secondary AML).

### 3.5 Thrombosis

A total of six patients suffered from thrombosis during the time between each sample collection. We wanted to confront those findings with conventional risk assessment which is aimed at evaluating the risk of thrombotic complications. In ET, one patient from the high-risk group (without a history of thrombosis) and three of the non-high-risk patients (two below the age of 60, and one above the age of 60 at the time of thrombosis) experienced thrombotic complications (two ischemic strokes, one myocardial infarction and one pulmonary embolism). In PV, two high-risk patients (one had an ischemic stroke at diagnosis) and none of the low-risk patients experienced thrombotic complications. Of those patients with thrombotic complications, only one had a history of thrombosis.

Two patients diagnosed with ET and initially assessed as low- or very-low-risk, experienced thrombosis despite still being below 60 at the time of the second sample collection (#34 and 28). In the first patient (male ET) who suffered from myocardial infarction at follow-up, we detected *ZRSR2* p.Ser447_Arg448dup c.1338_1343dup variant at high VAF (ranging 84,78% - 84,38% between samples), which is suspected to increase the risk of thrombosis. In the second patient (female ET), who experienced multiple pulmonary embolism events during the disease, we observed increasing VAF of *JAK2* p.Val617Phe driver mutation, from 7,14% at diagnosis to 43,32% at follow-up, despite being on cytoreductive treatment with hydroxyurea, later in combination with anagrelide.

## 4 Discussion

In addition to known driver mutations, NGS-based studies allow for detecting numerous variants in various genes. Those findings must be considered with caution because not all variants are confirmed to be pathogenic. Since the molecular landscape of MPNs is undiscovered area, we performed a thorough literature search to look for associations of detected variants with pathogenicity. The two most frequently detected variants - *CBL* p.Asp460del and *RUNX1* p.Glu422Ala - were described in various neoplasms, including hematologic, with no confirmed pathogenicity (9, 10). Additionally, the total number of mutations detected in 2^nd^ sample did not directly impact the disease’s phenotype, indicating the need for qualitative rather than a quantitative approach. On the other hand, our analysis allowed us to detect significant molecular changes in patients otherwise labeled as TN – one with *TP53* p.Arg248Leu – a widely described, pathogenic hotspot gain of function mutation– and *RUNX1* p.Arg423Gly variant - undescribed previously, here associated with apparent features of MPN (11). Additionally, we found two coexisting driver mutations in two patients – *JAK2* p.Val617Phe along with *MPL* p.Trp515Leu and *JAK2* p.Val617Phe with non-canonical *MPL* p.Ser505Asn. In both cases *MPL* variants had higher VAF than *JAK2* p.Val617Phe, and the latter *MPL* variant is confirmed pathogenic (4, 12). In another patient, we found a non-canonical *JAK2* p.Lys607Asn variant co-occurring with *JAK2* p.Val617Phe, which was described in AML patients (13). In a routine workup done with the PCR method, identifying true driver mutation in such patients is challenging, underlying the importance of complete, thorough molecular evaluation in MPN patients in the future.

Unsurprisingly, the current study’s two most frequently mutated genes were *TET2* and *DNMT3A*. Those mutations are widely described in hematologic neoplasms with inconsistent conclusions regarding their pathogenicity (14–17). Those and other mutations are also associated with clonal hematopoiesis of indeterminate potential (CHIP) - an effect of accumulation of specified mutations during life without a clear connection to morbidity (18). On the other hand, there are reports of increased genetic instability in *TET2-* or *DNMT3A-*mutated patients, possibly triggering further clonal hematopoietic expansion and contributing to acquiring additional HMR mutations (19–23). By analyzing the dynamics of those mutations in our study, we show that *TET2-*mutated patients are bearing this mutation from the diagnosis and acquisition of *DNMT3A* occurs later during the disease. While we did not find the exact correlations with those mutations with clinical phenotype, we confirmed that *TET2* and *DNMT3A* mutations detected in the first sample resulted in a significantly more mutations detected in the second sample.

In PV and ET, conventional risk assessment is aimed at predicting the risk of thrombosis - one of the factors significantly influencing the mortality and morbidity of patients diagnosed with MPN (7, 8). Here, thrombotic complications occurring after diagnosis were recorded in six patients, with the majority classified as non-high-risk. In older patients, accumulation of cardiovascular risk factors, age and history of thrombosis are helpful variables for predicting the risk of thrombosis. However, in younger patients, otherwise classified as low- or very-low-risk, data from consecutive genetic analyses may help to predict thrombotic complications correctly. In our study, two young patients experienced thrombotic complications, despite not harboring conventionally accepted risk factors. One had a significantly increasing *JAK2* p.Val617Phe allele burden, a process which was proven to be associated with the risk of thrombosis by Soudet et al (24). The second patient harbored *ZRSR2* p.Ser447_Arg448dup variant, which is likely benign when detected in MDS, but connected with splanchnic vein thrombosis in MPN (25, 26).

Wider accessibility to modern diagnostic methods initiated the search for different variables predicting the outcomes of patients with MPNs. In 2019 Tefferi et al. proposed a new prognostication system, incorporating results from genetic analyses, for ET and PV - MIPSS-ET and MIPSS-PV, respectively (5). However, the authors acknowledge the need for further evaluation of these systems. In our study, we applied the MIPSS scoring system based on the results of two consecutive samples from each patient. In as many as five ET patients, detected mutations allowed for the increase in MIPSS score, placing those patients in the high-risk group and suggesting the possible utility of this system in assessing the risk in a dynamic fashion. Additionally, we show the utility of MIPSS-ET in predicting the risk of sMF. On the other hand, one young PV patient with a detected pathogenic *TP53* mutation remained in the low-risk group according to MIPSS-PV. The appearance of high-risk variants at follow-up underlines the utility of sequential molecular evaluation of patients with MPNs.

As an exploratory objective, we used the MPN Prognostic Calculator created based on the work of Grinfeld et al. (4). Authors underline that the tool is rather a proposition and is not yet validated. This analysis showed significantly better prognosis across all evaluated scores for patients with PV compared to ET. It failed to distinguish patients with a high risk of developing sMF. Whether this is the result of the construction of the tool itself or the generalization of input genetic data, remains to be further investigated. Regarding MPNs, we encourage reporting and analyzing the impact of specific variants rather than stratifying the risk by the presence of mutation only.

Fibrotic or AML progression remains the most therapeutically challenging dilemma in PV and ET patients. Here, nine patients developed sMF during their disease. None of those patients displayed features of fibrosis on the initial bone marrow sample. However, the evaluation of the characteristics of the megakaryocytes in those samples might not be in accordance with the current knowledge, hence it cannot be ruled out that a proportion of those patients had an evolution from prefibrotic to an overt stage of MF. Nevertheless, we revealed that patients with fibrotic progression had a significantly higher mutational burden of their driver mutation at samples collected during the transformation compared to baseline and to patients without sMF. Additionally, those patients harbored specific variants previously attributed to fibrosis, including *ASXL1* p.Gly646TrpfsTer12, *RUNX1* p.Leu56Ser, *ZRSR2* p.Arg169Ter and *U2AF1* p.Gln157Pro (4, 16, 27–33). Apart from the abovementioned variants, other mutations were detected only in those patients who developed fibrosis but their importance requires further investigation (**Supplementary Table 2**). While the sample size is relatively small, those findings support the idea of repeated monitoring, allowing for the detection of increasing VAF and pathogenic variants before fibrotic progression while being less unpleasant for the patient and more objective than trephine biopsy.

In PV and ET, the interest in the correlation between treatment and mutational landscape is growing. In our study, we performed analyses on two homogenous group of patients – those treated with HU monotherapy and patients that endured without any treatment during the study. Based on our results, the potential leukemogenic effect of prolonged exposure to HU remains questionable, since those patients did not present features of expanding mutational landscape. It is unclear whether this result is an effect of treatment efficacy or rather phenotypically stable disease, not requiring treatment change. In treatment-free patients, detecting non-pathogenic variants and stable VAFs of driver mutations correlated with genetically stable disease and clinical picture. The question remains whether their stable molecular profile was the phenotype of the disease itself or, that by not introducing the treatment we did not expose the malignant clone to further genetic instability (34, 35).

In conclusion, we emphasize the need for careful interpretation of molecular findings, particularly the assessment of the pathogenicity of specific variants. Extensive studies evaluating the molecular profile of patients and confronting it with the diseases’ phenotype are critical. However, to date, only few studies evaluated the molecular findings in a dynamic fashion. Patients with MPNs must be evaluated more than once throughout their disease and it is insufficient to assess the risk only once, especially in young patients. Based on numerous studies across all fields of hematology, it is evident that diseases’ phenotype is driven and modulated by various molecular changes in addition to basic factors, such as age and history of thrombosis. This field is open for discovery, and authors of the current study believe that a larger portion of this knowledge remains unknown, including transcriptomics, epigenetic modifications, interaction with the microenvironment and paracrine activity of both malignant and non-malignant cells.

## Data availability statement

The data can be accessed by the following link: https://datadryad.org/stash/share/MSNwkQvXlqHqOWyTvAiizUBZBS6UBJZaxqSqwLXtHaM. 

## Ethics statement

The studies involving humans were approved by Bioethics Committee for Scientific Research, Medical University of Gdansk. The studies were conducted in accordance with the local legislation and institutional requirements. The participants provided their written informed consent to participate in this study.

## Author contributions

PS – conducted the research, recruited patients, analysed the data and written the manuscript. BW and MŻ – performed NGS analysis and reviewed the manuscript. AL – performed DNA isolation and reviewed the manuscript. MB – supervised the research, coordinated the work, helped writing and reviewed the manuscript.
